# Bioactive Compounds of *Plectranthus scutellarioides*: Extraction, Phytochemical Characterization and Biological Activities

**DOI:** 10.3390/molecules31183242

**Published:** 2026-09-14

**Authors:** Łukasz Kogut, Czesław Puchalski, Julia Jastrzębska, Grzegorz Zaguła

**Affiliations:** 1 Department of Bioenergetics, Food Analysis and Microbiology, Institute of Food Technology and Nutrition, Faculty of Technology and Life Science, University of Rzeszów, Aleja Rejtana 16C, 35-959 Rzeszów, Poland; lukasz.kogut2@wp.pl (Ł.K.); cpuchal@ur.edu.pl (C.P.); 2 Medical Centre in Łańcut LLC, Ignacego Paderewskiego 5, 37-100 Łańcut, Poland; j.jastrzebska2105@gmail.com

**Keywords:** *Plectranthus scutellarioides*, phytochemical variability, rosmarinic acid, abietane diterpenoids, extraction, ultrasound-assisted extraction, phytochemical characterization, biological activity, quantitative analysis, natural products

## Abstract

Background: *Plectranthus scutellarioides* is an ornamental and medicinal species of the Lamiaceae family characterized by substantial phytochemical diversity and variability. This review critically synthesizes current evidence on its bioactive compounds, factors determining phytochemical composition, extraction efficiency, analytical characterization, and biological activity, with particular emphasis on relationships between plant material, extraction conditions, metabolite profiles, and experimentally determined bioactivity. Methods: A narrative review of the available scientific literature was conducted using studies addressing the phytochemical composition of *P. scutellarioides*, cultivar and cultivation-related variability, extraction procedures, analytical characterization, and biological activities of extracts and isolated metabolites. Particular attention was given to quantitative phytochemical and biological data and to methodological factors limiting direct comparison among studies. Results: *P. scutellarioides* contains phenolic acids, flavonoids, anthocyanins, abietane-type diterpenoids, and volatile constituents, with rosmarinic acid representing a major phytochemical marker. Substantial quantitative variability is associated with cultivar, geographical origin, cultivation conditions, plant material, and extraction procedure. Optimized ultrasound-assisted extraction yielded 110.8 ± 4.5 mg g^−1^ DW of rosmarinic acid after 45 min, approximately twice the recovery obtained by conventional heat-reflux extraction over the same period. Quantitative profiling also demonstrated marked cultivation-dependent changes in volatile metabolites. Biological studies revealed assay- and compound-dependent antioxidant, antimicrobial, anti-inflammatory, and cytotoxic/antiproliferative activities. In particular, isolated diterpenoids inhibited NF-κB activity with IC_50_ values of 4.5–11.2 µM, providing compound-specific quantitative evidence of anti-inflammatory potential. However, differences in extract composition, experimental models, and assay conditions, together with limited standardization and the predominance of in vitro studies, substantially restrict cross-study comparisons and assessment of translational relevance. Conclusions: The available evidence indicates that the biological potential of *P. scutellarioides* should be interpreted through an integrated extraction–composition–activity framework rather than from the simple presence of individual metabolites. Future studies should prioritize standardized and botanically characterized plant material, clearly defined harvesting conditions, quantitative phytochemical characterization, comparable biological endpoints, bioactivity-guided identification of active constituents, and in vivo validation. Establishing reproducible relationships between plant material, extraction conditions, chemical composition, and biological effects will be essential for assessing the pharmaceutical, cosmetic, and functional potential of this species.

## 1. Introduction

*Plectranthus scutellarioides* (L.) R.Br. is a species belonging to the family Lamiaceae, widely recognized as an ornamental plant because of its remarkable morphological diversity and the wide range of leaf coloration [1,2,3,4,5]. In the scientific literature, this species is also referred to by numerous taxonomic synonyms, the most common being *Coleus blumei*, *Coleus scutellarioides*, and *Solenostemon scutellarioides* [1,5,6,7]. This nomenclatural diversity is important when reviewing the literature because studies concerning phytochemical composition, extraction methods, and biological activity may be published under different names referring to the same species [5,7,8,9]. Contemporary systematic classifications increasingly recommend the use of the name *Coleus scutellarioides* (L.) Benth.; however, the name *Plectranthus scutellarioides* remains widely used in pharmacognostic and phytochemical literature [4,5,7].

The importance of this species extends beyond its ornamental value [3,4,10,11,12]. *P. scutellarioides* is a rich source of numerous secondary metabolites, among which phenolic acids, flavonoids, anthocyanins, diterpenoids, and other biologically active compounds are considered particularly important [6,12,13,14,15]. These compounds have attracted considerable attention because of their potential antioxidant, antimicrobial, anti-inflammatory, and cytotoxic properties [8,13,14,16]. At the same time, the chemical composition of the plant may vary depending on the cultivar, origin of the plant material, environmental conditions, plant organ, and extraction method [6,17,18,19]. Consequently, studies on *P. scutellarioides* increasingly focus not only on identifying individual metabolites but also on evaluating the relationships between extraction procedures, phytochemical profiles of the resulting extracts, and their biological activities [6,12,18,19].

### 1.1. Botanical Characteristics

*P. scutellarioides* is an evergreen perennial herb or subshrub [1,2,20,21,22]. The plant usually reaches approximately 0.5–1.0 m in height, although under favorable conditions it may grow up to about 2 m [1,22]. The stems are soft, succulent, relatively brittle, and exhibit the characteristic quadrangular cross-section typical of members of the Lamiaceae family [4,20,23]. The species displays remarkable morphological variability, particularly in its leaves, which differ among cultivars in size, shape, surface texture, and coloration intensity [2,4,24,25,26].

Leaves are the most characteristic morphological feature of the plant and are also the most frequently used material in phytochemical studies [8,17,19]. They may be ovate, cordate, or elliptical, with margins that are typically crenate, serrated, or dentate [4,20,27,28]. The leaf blade may be either monochromatic or multicolored, displaying shades of green, red, pink, purple, yellow, and brown [4,19,22,28]. Distinct pigmentation patterns may occur within the same plant, representing one of the principal features distinguishing individual cultivars [2,5,21,28,29].

Leaf pigmentation is important not only from an ornamental perspective but also from a phytochemical standpoint [4,6]. The intense red and purple coloration is associated with the presence of anthocyanin pigments, whereas the relative proportions of individual pigments may differ among cultivars [4,6,19,28]. This variability may contribute to differences in the overall chemical profile and antioxidant potential of the plant material [10,19]. Consequently, results obtained from one cultivar of *P. scutellarioides* cannot always be directly generalized to the entire species [6].

The flowers of *P. scutellarioides* are small, usually white, bluish, or violet, and possess the typical bilabiate structure characteristic of the Lamiaceae family [15,17,22,24,27,30]. They are arranged in elongated spike-like inflorescences [22,27,30]. Although most studies on the chemical composition of *P. scutellarioides* have focused on the leaves, other plant organs, including stems and roots, may also serve as valuable sources of biologically active compounds [14].

Microscopic studies of the leaves have revealed structures typical of the Lamiaceae family, including epidermal trichomes and diacytic stomata [4,29]. Trichomes may play an important role in the biosynthesis, storage, and secretion of certain secondary metabolites [28,29]. Therefore, the anatomical characteristics of the plant may provide valuable complementary information to phytochemical analyses, particularly in studies investigating the localization of specific metabolite groups within individual tissues [29].

### 1.2. Distribution and Geographical Occurrence

*P. scutellarioides* is primarily associated with tropical and subtropical regions [2,8,15,17,28]. Its principal center of origin is considered to be the Paleotropical region, particularly Southeast Asia, including the Malay Archipelago, Indonesia, Malaysia, and the Philippines, as well as other parts of Asia and northern Australia [2,4,28]. Owing to its long history of cultivation and popularity as an ornamental plant, the species has also been introduced into many other regions of the world [2,5,9].

At present, *P. scutellarioides* is cultivated or naturalized throughout numerous tropical and subtropical regions of Africa and the Americas [9,12,15,17]. It is particularly widespread in regions characterized by warm and humid climates [2,21]. In Europe and other temperate regions, it is cultivated primarily as an ornamental species [2,3,15].

Under natural and semi-natural conditions, the species prefers moist, warm, and partially shaded habitats [2,4,21]. It commonly occurs along forest margins, in shrublands, near rivers and streams, and in anthropogenically disturbed environments [2,9,21]. Its ability to thrive in diverse habitats reflects its considerable ecological plasticity [2,21].

Environmental conditions may also influence the phytochemical composition of the plant [10,17]. Light intensity, temperature, water availability, soil mineral composition, altitude, and environmental stress factors may affect the biosynthesis of secondary metabolites [2,17,31,32]. Consequently, plant material collected from different geographical regions may differ in the content of phenolic acids, flavonoids, anthocyanins, and other bioactive constituents [4,17]. This variability should be taken into account when comparing phytochemical and biological studies performed using plant material from different geographical origins [17].

## 2. Materials and Methods

The present narrative review was prepared based on scientific literature identified through a structured search of the PubMed/MEDLINE, Scopus, Web of Science, and Google Scholar databases. The literature search was completed on 31 July 2026. Because the study was designed as a narrative rather than a systematic review, a formal PRISMA-style record-flow procedure was not prospectively implemented, and exact numbers of records retrieved and screened at each stage were not recorded. Therefore, these values are not reported retrospectively in order to avoid presenting potentially inaccurate estimates. Publications published primarily between 2000 and 2026 were considered, with particular emphasis placed on studies investigating the phytochemistry, extraction methods, analytical characterization, and biological activities of *Plectranthus scutellarioides* (syn. *Coleus scutellarioides*). Because this species has been reported under different taxonomic names, the literature search also included its commonly used synonyms. The search strategy combined taxonomic terms using the Boolean operator OR and subsequently linked them with thematic search terms using AND. The principal search structure was: (“*Plectranthus scutellarioides*” OR “*Coleus scutellarioides*” OR “*Coleus blumei*”) AND (phytochemistry OR “phytochemical profile” OR “bioactive compounds” OR “phenolic acids” OR “rosmarinic acid” OR flavonoids OR anthocyanins OR diterpenoids OR “abietane diterpenoids” OR “essential oils” OR extraction OR “ultrasound-assisted extraction” OR “microwave-assisted extraction” OR HPLC OR “LC-MS/MS” OR “GC-MS” OR NMR OR “antioxidant activity” OR “antimicrobial activity” OR “anti-inflammatory activity” OR “cytotoxic activity” OR “pharmacological activity” OR metabolomics OR “natural products”). The search syntax was adapted, when necessary, to the indexing and search functionalities of the individual databases. The reference lists of eligible publications were additionally screened to identify further relevant studies.

Original research articles and review papers were included when they provided information on the phytochemical composition of *P. scutellarioides*, extraction procedures, analytical methods used for metabolite identification and quantification, or the biological activities of plant extracts and isolated compounds. Full-text articles published in English were considered eligible. Publications focusing on closely related *Plectranthus* species were included only when they provided complementary information relevant to compounds also identified in *P. scutellarioides* or contributed to the interpretation of phytochemical or pharmacological findings. Conference abstracts without full-text availability, editorials, duplicate records, and publications not directly related to the objectives of this review were excluded.

The selected publications were subjected to qualitative analysis according to the predefined thematic scope of the review. Information was extracted on botanical characteristics, geographical distribution, major classes of secondary metabolites, extraction techniques, analytical methodologies, and reported biological activities, including antioxidant, antimicrobial, anti-inflammatory, and cytotoxic effects. Particular attention was given to factors influencing phytochemical variability, including cultivar differences, environmental conditions, extraction procedures, and analytical approaches, as well as to current limitations of the available evidence and future research perspectives. Information on the harvest period, developmental stage, and plant part was also considered when reported; however, these variables were inconsistently documented in the available original studies, which limited their systematic comparison across the literature.

As this study was conducted as a narrative literature review and did not involve human participants, identifiable personal data, or experimental procedures, approval from an ethics committee and study registration were not required.

## 3. Bioactive Compounds and Their Phytochemical Characteristics

The phytochemical composition of *P. scutellarioides* shows considerable variability depending on genotype, geographical origin, cultivation conditions, and environmental factors. To facilitate comparison among studies, the available evidence concerning the influence of cultivar, plant origin, and cultivation or collection conditions on the major metabolite groups is summarized in Table 1. Such variability is particularly relevant for phenolic compounds, including rosmarinic acid, flavonoids, and anthocyanins, and should be considered when comparing phytochemical profiles obtained in independent studies.

### 3.1. Phenolic Acids

Phenolic acids represent one of the most important groups of bioactive compounds identified in *P. scutellarioides* and contribute substantially to the antioxidant potential of its extracts [34,35,36,37,38,39,40,41]. However, their composition and concentration may vary depending on the origin of the plant material and the extraction procedure employed [10,17,42]. Solvent polarity, solvent-to-solid ratio, extraction temperature and time, as well as sample preparation and storage conditions are particularly important factors affecting extraction efficiency [10,17,19,42].

Rosmarinic acid is the principal phenolic compound identified in the leaves of *P. scutellarioides*. It is an ester of caffeic acid and 3,4-dihydroxyphenyllactic acid [17,37]. As a characteristic metabolite of many members of the Lamiaceae family, its high abundance makes it an important phytochemical marker of this species [17,24,34,36]. Quantification of rosmarinic acid is valuable for comparing cultivars and extracts and may support future standardization efforts [17,36]. Nevertheless, reported concentrations differ considerably depending on the extraction method and the way results are expressed [9,17].

Quantitative studies confirm that rosmarinic acid recovery from *P. scutellarioides* is strongly dependent on extraction conditions. In particular, optimized ultrasound-assisted extraction has been shown to provide substantially higher recovery than conventional heat-reflux extraction, demonstrating the importance of extraction strategy for the quantitative phytochemical profile of this species [12]. A detailed quantitative comparison of these extraction procedures is provided in Section 4.1.

The presence of multiple hydroxyl groups enables rosmarinic acid to donate hydrogen atoms and stabilize free radicals, accounting for its strong antioxidant activity [37,43,44,45]. It has also been investigated for its potential antimicrobial, anti-inflammatory, and cytoprotective properties [37,46,47]. In *P. scutellarioides*, rosmarinic acid may substantially contribute to the biological activity of extracts, although the observed effects are likely the result of the combined action of multiple metabolites [9,48,49].

Besides rosmarinic acid, caffeic, chlorogenic, gallic, and *p*-coumaric acids have also been identified [9,44]. Caffeic acid serves as a precursor in the biosynthesis of more complex phenolic metabolites, including rosmarinic acid, whereas chlorogenic acid, an ester of caffeic and quinic acids, is a widely distributed plant antioxidant [37,46]. The occurrence of gallic and *p*-coumaric acids further increases the diversity of the phenolic fraction [48].

Other noteworthy metabolites include nepetoidin A and nepetoidin B, derivatives of nepetoidic acid characteristic of Lamiaceae species [37,43,47]. Their presence indicates that the phenolic fraction of *P. scutellarioides* is considerably more complex than would be suggested solely by the predominance of rosmarinic acid [10,34,48].

### 3.2. Flavonoids

Flavonoids constitute an important group of secondary metabolites in *Plectranthus scutellarioides*, performing protective functions against UV radiation, oxidative stress, and adverse environmental conditions [10,19,34]. They occur both as free aglycones and as numerous glycosylated derivatives, with glycosylation influencing their solubility, stability, and extraction efficiency [34,37].

An important subgroup comprises quercetin derivatives, including quercetin 3-glucoside, quercitrin, and more complex glycosylated and acetylated derivatives [4,11]. Free quercetin has also been detected in extracts, although its reported concentration depends on the extraction and analytical methods employed [10,11]. The diversity of these compounds reflects the extensive flavonoid metabolism of this species [9,10,50].

Apigenin and luteolin derivatives, including glucuronidated, glycosylated, and acetylated forms, have also been identified [10,15,34]. Both apigenin and luteolin are widely studied flavonoids with potential antioxidant, anti-inflammatory, and antimicrobial properties [37,51].

Chromatographic analyses have further revealed the presence of rutin, catechin, isoquercitrin, kaempferol, and their glycosylated derivatives [9,48,49]. The pronounced structural diversity of flavonoids may influence the biological activity of extracts, as these compounds differ in stability, bioavailability, and biological effects, while their coexistence may result in additive or synergistic interactions [1,37,48].

Due to the relatively high polarity of many flavonoid glycosides, extraction is typically performed using water, methanol, ethanol, or aqueous-organic solvent mixtures [4,37]. Solvent composition can selectively influence the phytochemical profile of the obtained fraction and should therefore be considered when comparing the composition and biological activity of different extracts [9,19].

Quantitative analyses confirm substantial variation in flavonoid content among *P. scutellarioides* cultivars and extraction procedures. In a comparative study of six cultivars, total flavonoid content ranged from 1.667 to 4.305 mg RE g^−1^ DWE following maceration and from 2.222 to 4.422 mg RE g^−1^ DWE following Soxhlet extraction [10]. The cultivar ‘Trailing Psycholeus’ showed the highest flavonoid content after maceration, whereas ‘Flamingo’ showed the highest value after Soxhlet extraction [10]. These findings demonstrate that both cultivar and extraction procedure contribute to the quantitative variability of the flavonoid fraction and should be considered when comparing phytochemical profiles across studies.

### 3.3. Anthocyanins

Anthocyanins are among the most characteristic metabolites of *Plectranthus scutellarioides*, being responsible for the diverse leaf coloration ranging from green and pink to deep red, purple, and reddish-brown [24,37,52]. These differences result from both the qualitative and quantitative composition of pigments and their distribution within plant tissues [7,24,38,53].

Chemically, anthocyanins are glycosylated and frequently acylated derivatives of anthocyanidins [34]. Cyanidin derivatives appear to represent the predominant anthocyanin group reported in *P. scutellarioides*. Detailed HPLC analyses identified three cyanidin derivatives containing one *p*-coumaroyl residue and two hexose residues, two of which were additionally malonylated; the malonylated cyanidin glycosides occurred in higher amounts [34]. Cyanidin 3,5-diglucoside acylated with *p*-coumaric acid has also been reported in the leaves [34]. Pelargonidin derivatives have additionally been identified in *P. scutellarioides* [40]. However, directly comparable quantitative concentration ranges for individual anthocyanins across cultivars with different leaf colors remain insufficiently reported, preventing a reliable quantitative ranking among differently pigmented cultivars.

The anthocyanin profile varies substantially among *P. scutellarioides* cultivars and is closely associated with leaf pigmentation [10,35]. Intensely red and purple phenotypes generally exhibit greater anthocyanin accumulation than predominantly green-leaved forms [34,35]. In addition to cultivar-dependent differences, anthocyanin accumulation is strongly affected by environmental conditions. Experimental cultivation under different light sources resulted in significant variation in anthocyanin content, with the highest content per gram of dry weight observed under ceramic discharge metal-halide (CDM) illumination [34]. Thus, both genotype and cultivation conditions should be considered when comparing anthocyanin levels among differently colored cultivars. Nevertheless, standardized quantitative concentration ranges for individual anthocyanins across green-, red-, and purple-leaved *P. scutellarioides* cultivars are currently unavailable.

Anthocyanin accumulation is strongly influenced by environmental conditions, particularly light intensity and spectral composition [34]. Their concentration is also affected by temperature, mineral nutrient availability, and abiotic stress, including water deficit and exposure to certain metals [34,48].

Beyond their pigmentary role, anthocyanins also participate in antioxidant reactions and may contribute substantially to the antioxidant capacity of extracts obtained from intensely pigmented leaves [40]. However, their relatively low stability under elevated temperatures and unfavorable pH conditions represents a major challenge during extraction, purification, and storage [10,40].

### 3.4. Diterpenoids

Diterpenoids are among the most characteristic and structurally diverse metabolites of the genus *Plectranthus* [24,37,52]. Of particular importance are abietane-type derivatives, whose diversity arises from differences in oxidation state, the presence of various functional groups, and modifications of the diterpene skeleton [7,24,38,53].

Among the noteworthy compounds isolated from *P. scutellarioides* are spiroscutelones A, B, and C, which are abietane-type diterpenoids with unusual chemical structures [10,27]. Spiroscutelone A is especially distinctive because it contains a rare cyclobutane ring system [11,27]. These compounds are of considerable interest in chemotaxonomy and natural products chemistry, as well as in the search for novel bioactive molecules [7,27,38,53].

Available isolation studies provide quantitative evidence that these diterpenoids occur at relatively low recoverable levels in *P. scutellarioides*. From 300 g of dried leaves, 25.2 g of chloroform extract was obtained, from which 2.0 mg of spiroscutelone A, 6.0 mg of spiroscutelone B, and 5.1 mg of spiroscutelone C were subsequently isolated [11]. In another study, 0.93 g of a diterpenoid-enriched dichloromethane extract was obtained from 0.8 kg of fresh aerial parts, yielding 2.8 mg of coleon O, 0.5 mg of coleon G, and 0.6 mg of lanugone K after chromatographic purification; other isolated diterpenoids were generally recovered in amounts of approximately 0.7–3.1 mg [13]. Additionally, 8 mg of a new abietane-type diterpenoid glucoside was isolated from an ethyl acetate fraction obtained from 420 g of aerial plant material [15]. These results indicate that, although structurally diverse diterpenoids are characteristic constituents of *P. scutellarioides*, individual compounds are generally recovered in milligram quantities, and direct comparison of their abundance should be interpreted cautiously because the studies differed substantially in plant material, solvent system, extraction procedure, and fractionation strategy [11,13,15].

Coleons and other abietane derivatives have also been identified in this species, including coleon O, coleon G, fredericone derivatives, and compounds designated as scutellarioidolides and scutellarioidones [7,10,54,55]. Even subtle structural differences may substantially influence their biological activity, while the presence of hydroxyl, carbonyl, quinone, and acetyl groups determines their chemical reactivity and interactions with cellular components [37,38,56,57].

Interest in *Plectranthus* diterpenoids is primarily driven by the cytotoxic and antiproliferative activities exhibited by some of these compounds in experimental studies [10,37,54,58,59,60]. However, results obtained from in vitro studies should be regarded mainly as a basis for further mechanistic investigations and should not be interpreted as evidence of confirmed therapeutic efficacy [15,37,59,61,62].

**Figure 1:**
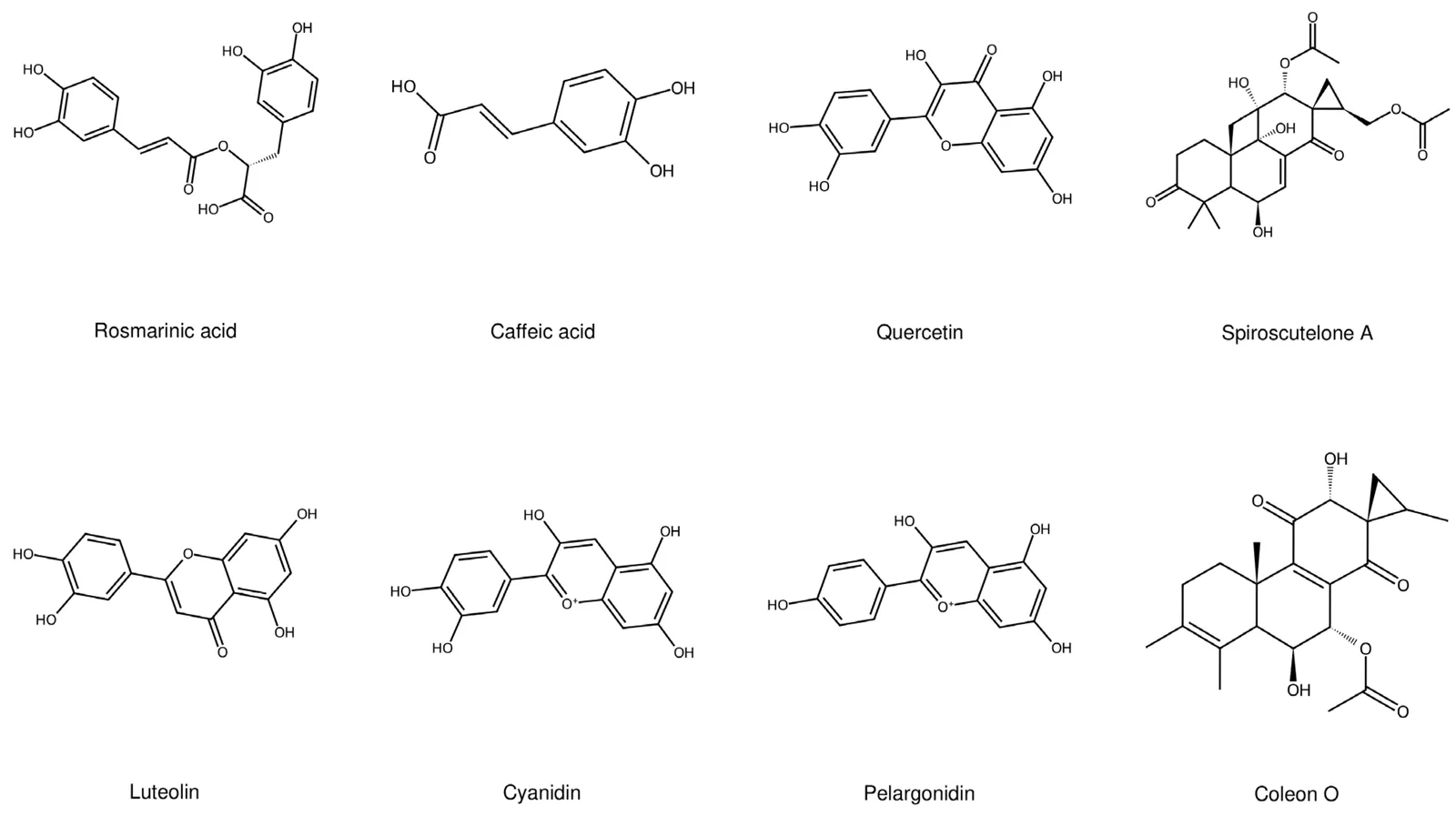
Chemical structures of representative bioactive metabolites reported in *Plectranthus scutellarioides*.

The structural diversity of the principal bioactive metabolites reported in *P. scutellarioides* is illustrated in Figure 1. Representative compounds include phenolic constituents such as rosmarinic and caffeic acids, flavonoids such as quercetin and luteolin, the anthocyanidin aglycones cyanidin and pelargonidin, and structurally more complex diterpenoids represented by spiroscutelone A and coleon O. These structures illustrate the substantial chemical diversity of the species, ranging from highly hydroxylated polar phenolics to less-polar polycyclic diterpenoids.

Coleon O and spiroscutelone A were redrawn from the original isolation studies [13,27], with stereochemical notation retained as depicted in those sources. Cyanidin and pelargonidin are shown as flavylium cations.

### 3.5. Essential Oils and Other Secondary Metabolites

The volatile fraction of *Plectranthus scutellarioides* constitutes a relatively small proportion of the chemical composition of the leaves but exhibits considerable qualitative diversity [1,35,63]. Its composition depends on the genotype, cultivation site and conditions, developmental stage of the plant, and the isolation method employed, resulting in substantial variation among published studies [9,35,42,63,64,65].

GC-MS analyses have demonstrated the presence of sesquiterpenes and their oxygenated derivatives, including spathulenol, germacrene D, bicyclogermacrene, and trans-caryophyllene [1,61,66]. These compounds are commonly found in the essential oils of Lamiaceae species and may contribute to the antimicrobial and antioxidant properties of the volatile fraction [1,24,35].

More recent quantitative profiling of volatile compounds in *C. scutellarioides* ‘Electric Lime’ further demonstrated the strong influence of cultivation conditions on the volatile fraction. In the original study, in vivo material referred to the conventionally grown source plant from which the in vitro cultures were initiated, whereas in vitro material referred to plantlets maintained under controlled tissue-culture conditions [34]. Thirty compounds were identified in the in vivo plant material, compared with 33 compounds in plantlets derived from in vitro cultures. The total volatile-compound content was 2848.59 µg g^−1^ in the in vivo material and increased to 8191.47 µg g^−1^ in control in vitro cultures without phytohormone supplementation. Among the predominant constituents, 1-octen-3-ol accounted for 14.44% of the volatile fraction in vivo and 47.35% under control in vitro conditions, while copaene decreased from 18.44% to 5.36% and α-pinene from 15.63% to 6.87%, respectively. The corresponding proportions of α-selinene were 7.76% and 5.70%, whereas 3-octanol increased from 4.79% to 15.19%. These results demonstrate that cultivation conditions can markedly alter both the total abundance and relative composition of volatile metabolites in *C. scutellarioides*. Volatile compounds were profiled using solid-phase microextraction coupled with GC-MS (SPME-GC-MS), providing quantitative characterization of the volatile fraction without conventional essential-oil isolation. Therefore, an essential-oil yield cannot be directly calculated from these data [34].

Phytol, a diterpene alcohol associated with chlorophyll metabolism, has also been identified in lipophilic extracts [1,35,42]. Its relatively high abundance in certain chromatographic profiles may result from extraction procedures favoring nonpolar compounds [35]. It is important to distinguish the composition of the genuine essential oil from the overall profile of GC-MS-analyzed extracts, which may also contain less volatile compounds and their degradation products [10,35].

The phytochemical profile further includes triterpenoids such as oleanolic, ursolic, and betulonic acids, as well as α- and β-amyrin [19,35,37,57]. These compounds have attracted attention because of their potential anti-inflammatory, antimicrobial, and cytotoxic properties [37,67]. Phytosterols, including β-sitosterol, stigmasterol, and campesterol, have also been detected, primarily in less polar extract fractions [35,37,53].

Preliminary phytochemical screening has additionally indicated the presence of saponins, tannins, alkaloids, and quinones [4,19,35]. However, for these groups, available data are often qualitative in nature, and the identification of individual compounds remains limited [4]. Consequently, the results of simple phytochemical screening tests should be confirmed using more selective instrumental analytical techniques [1,4].

**Figure 2:**
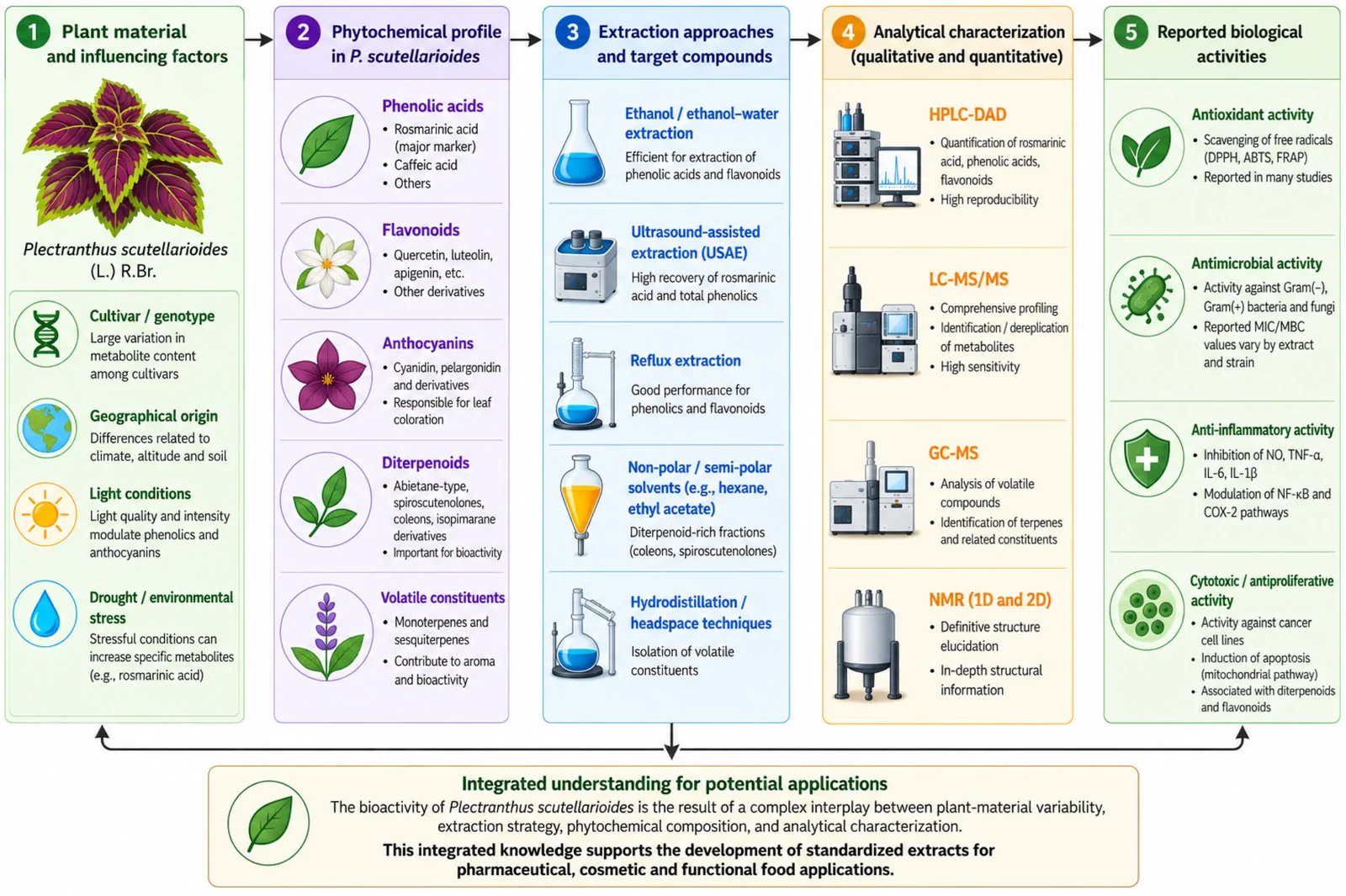
Integrated overview of the relationships between plant-material variability, phytochemical profile, extraction approaches, analytical characterization, and reported biological activities of *Plectranthus scutellarioides*.

The relationships between plant-material variability, phytochemical composition, extraction strategy, analytical characterization, and the reported biological activities of *P. scutellarioides* are summarized in Figure 2. This integrated framework highlights how differences in plant origin and cultivation conditions, together with the applied extraction and analytical procedures, may influence the chemical profile and consequently the observed biological effects.

Figure 2 was prepared with the assistance of an artificial intelligence-based tool; all graphical elements and scientific content were subsequently reviewed and verified by the authors.

## 4. Extraction and Analysis of Bioactive Compounds

Extraction methodology is a major source of variability in the phytochemical profiles reported for *P. scutellarioides* [6,37,68]. This is particularly relevant because the species contains chemically diverse metabolites ranging from polar phenolic acids and flavonoid glycosides to less-polar diterpenoids, triterpenoids, and volatile constituents [35,37,64,68]. Consequently, extraction conditions determine not only the overall recovery of secondary metabolites but also the relative representation of individual compound classes in the resulting extracts. The composition of the obtained extract depends primarily on solvent properties, extraction conditions, and sample preparation, while the extraction strategy should be selected according to the physicochemical properties of the target metabolites. In *P. scutellarioides*, this methodological variability is particularly important when comparing the recovery of major phytochemical markers, including rosmarinic acid, across individual studies. Therefore, the extraction methods discussed below are evaluated primarily in relation to their application to *P. scutellarioides* and their influence on the resulting phytochemical profile [9,17,37,69].

### 4.1. Extraction Methods

Maceration has been applied to *P. scutellarioides* using water, ethanol, methanol, and their mixtures to obtain extracts containing phenolic compounds, flavonoids, anthocyanins, and other secondary metabolites. Although this conventional approach may limit thermal degradation of temperature-sensitive constituents, studies on *P. scutellarioides* demonstrate that the number and relative abundance of compounds recovered by maceration depend strongly on solvent composition and cannot be interpreted as a direct measure of total extraction efficiency. Thus, maceration remains useful for broad phytochemical extraction, but its relatively long processing time and high solvent consumption limit its efficiency compared with assisted extraction approaches [6,10,40,70,71].

Ultrasound-assisted extraction (UAE) has shown particular relevance for the recovery of phenolic constituents from *P. scutellarioides*, especially rosmarinic acid, one of the principal phytochemical markers of this species. Its extraction efficiency is strongly influenced by solvent composition, ultrasound conditions, temperature, extraction time, and the solvent-to-solid ratio. Importantly, quantitative studies demonstrate that optimization of these parameters can substantially increase rosmarinic acid recovery while shortening extraction time compared with conventional procedures [12,17,72].

Solvent composition is a critical determinant of the phytochemical profile obtained from *P. scutellarioides*. Ethanol, methanol, and their aqueous mixtures have been used for the recovery of phenolic constituents, although their extraction selectivity differs according to solvent polarity. In particular, high ethanol concentrations have been associated with efficient recovery of rosmarinic acid, whereas the addition of water may increase the extraction of more polar constituents. Consequently, differences in solvent composition should be considered when comparing the qualitative and quantitative phenolic profiles reported for *P. scutellarioides*, as extracts obtained using different solvent systems are not directly equivalent [6,17,68,72].

Microwave-assisted extraction (MAE) has also been applied to *P. scutellarioides*, particularly for the recovery of water-soluble constituents. Its effectiveness depends on microwave power, extraction temperature, and exposure time, which influence both extraction efficiency and metabolite stability. Although increased extraction intensity can accelerate metabolite recovery, excessive thermal exposure may promote degradation of phenolic compounds, pigments, and other thermolabile constituents. Therefore, in *P. scutellarioides*, MAE should be considered primarily as a rapid extraction approach whose performance depends on balancing metabolite recovery with the stability of the targeted phytochemical fraction [6,70,72].

Soxhlet extraction has been used in phytochemical studies of *P. scutellarioides* to obtain fractions differing in polarity and chemical composition. Sequential extraction with solvents of increasing polarity, beginning with non-polar solvents such as hexane, enables separation of lipophilic constituents from more polar metabolite fractions and facilitates subsequent association of particular phytochemical groups with biological activity. However, because Soxhlet extraction involves prolonged exposure to elevated temperatures, it is less suitable for thermolabile constituents such as anthocyanins, selected phenolic compounds, and volatile metabolites. Its application to *P. scutellarioides* is therefore more appropriate when targeting relatively stable medium- and low-polarity constituents [10,38,40,57,67,68,69,70,71,72,73,74,75].

Studies employing reflux extraction, infusion, decoction, and turbo-extraction further demonstrate the methodological heterogeneity of the available evidence on *P. scutellarioides*. Because these procedures differ substantially in solvent composition, temperature, extraction time, and extraction intensity, the resulting phytochemical profiles cannot be treated as directly equivalent. This limitation is particularly relevant when biological activities are compared across studies without simultaneous quantitative characterization of the corresponding extracts. At present, the available evidence is insufficient to determine whether differences in biological activity among extracts produced by these procedures primarily reflect extraction efficiency, selective recovery of particular metabolites, degradation of thermolabile constituents, or genuine biological variability of the investigated plant material [69,76]. Future comparative studies should therefore characterize extracts quantitatively under standardized conditions before attributing differences in biological activity to the extraction procedure itself.

Modern optimization of extraction processes increasingly incorporates the principles of green chemistry, aiming to reduce the use of toxic solvents, energy consumption, and extraction time [17,70]. Accordingly, ultrasound-assisted and microwave-assisted extraction, together with the use of ethanol, water, and their mixtures as environmentally friendly extraction solvents, are regarded as particularly promising approaches [70].

Quantitative comparison with conventional heat-reflux extraction further demonstrates the higher efficiency of UAE for rosmarinic acid recovery. Under optimized conditions (100% ethanol, 30 kHz, 45 min), UAE yielded 110.8 ± 4.5 mg RA g^−1^ DW, compared with 55.6 ± 3.1 mg g^−1^ DW obtained by conventional heat-reflux extraction after the same 45 min extraction period. Thus, UAE provided approximately twice the rosmarinic acid recovery within the same extraction time [12].

Solvent composition also markedly affected rosmarinic acid recovery. When aqueous ethanol concentrations ranging from 0 to 100% (v/v) were compared under otherwise constant extraction conditions, recovery increased with increasing ethanol concentration and reached 82.1 ± 6.5 mg g^−1^ DW with pure ethanol [12]. Subsequent optimization of ethanol concentration, ultrasound frequency, and extraction duration further improved extraction efficiency, confirming the importance of combined process optimization [12].

In contrast to rosmarinic acid, direct quantitative comparisons of diterpenoid extraction efficiency and extraction kinetics using different solvents and extraction technologies remain scarce for *P. scutellarioides*. Available studies have primarily focused on the isolation and structural characterization of individual diterpenoids using conventional solvent extraction followed by chromatographic fractionation rather than systematic comparisons of extraction rates and recovery yields [13,15]. Therefore, the currently available evidence does not allow a reliable quantitative ranking of extraction methods for diterpenoids. Future studies should therefore conduct controlled head-to-head comparisons of different extraction methods using the same plant material and standardized conditions, with quantitative determination of individual diterpenoid recovery.

Overall, the available quantitative evidence demonstrates that extraction methodology is not merely a technical step but a major determinant of the phytochemical profile reported for *P. scutellarioides*. This is particularly evident for rosmarinic acid, for which optimized UAE provides substantially greater recovery within shorter extraction times than conventional heat-reflux extraction [12]. In contrast, comparable quantitative datasets are not yet available for most diterpenoids, anthocyanins, and other individual metabolites. Consequently, the current literature permits quantitative comparison of extraction efficiency for selected phytochemical markers, particularly rosmarinic acid, but does not support generalization of these findings to the complete metabolite profile of the species.

### 4.2. Analytical Techniques (HPLC, LC-MS/MS, GC-MS, and NMR)

Analytical studies of *P. scutellarioides* differ substantially in both their objectives and the depth of phytochemical characterization achieved. Screening-oriented investigations based on TLC and spectrophotometric assays provide information on major phytochemical classes or aggregate parameters, whereas HPLC- and LC-MS-based studies enable more selective identification and, when appropriate standards are available, quantitative determination of individual metabolites [4,32,33,39,49]. More advanced LC-MS/MS and NMR approaches have additionally enabled the characterization and structural elucidation of complex metabolites, including flavonoid derivatives and abietane-type diterpenoids [13,15,27]. Consequently, differences among published phytochemical profiles may reflect not only genuine biological variability but also differences in extraction procedures and analytical resolution. Results obtained using total phenolic or flavonoid assays should therefore not be directly equated with compound-specific chromatographic quantification. This methodological heterogeneity remains an important limitation for inter-study comparisons and highlights the need to combine qualitative identification with quantitative determination of major phytochemical markers whenever possible [10,15,27,33,49]. Representative analytical strategies applied to the principal metabolite groups are summarized in Table 2.

A clear distinction should be made between qualitative identification and quantitative determination. Qualitative analysis aims to establish which metabolites are present and may rely on chromatographic retention behavior, UV-Vis spectra, accurate molecular mass, isotope patterns, and MS/MS fragmentation. Identification confidence increases when several orthogonal parameters agree and is highest when the analyte is compared directly with an authentic standard. Quantitative analysis, in contrast, requires measurement of analyte concentration using validated calibration procedures, preferably with authentic reference standards, and appropriate assessment of analytical performance. Therefore, the detection of an ion or chromatographic peak consistent with a given metabolite should not, by itself, be interpreted as quantitative determination or unequivocal structural confirmation.

Accordingly, metabolites reported solely on the basis of qualitative screening or tentative spectral annotation should be regarded as a lower level of analytical evidence than compounds confirmed and quantitatively determined using validated chromatographic methods with authentic reference standards.

Dereplication based on LC-MS/MS or GC-MS spectral data also carries a risk of false-positive metabolite annotation, particularly in chemically complex plant extracts containing isomeric or structurally related compounds. A match between an experimental mass spectrum and a spectral database or library should therefore be regarded as tentative identification rather than conclusive structural confirmation when no authentic reference standard is available. High-confidence identification of known metabolites requires agreement with an authentic standard whenever possible, including appropriate chromatographic and spectral characteristics, whereas definitive characterization of previously unknown or structurally ambiguous compounds generally requires compound isolation and NMR-based structural elucidation in combination with high-resolution MS data.

HPLC has been extensively applied to the characterization of polar and moderately polar metabolites in *P. scutellarioides*, particularly phenolic acids and flavonoids. Rosmarinic acid represents one of the principal quantitative phytochemical markers, allowing differences associated with extraction procedures, cultivation conditions, and plant-material standardization to be evaluated. Simultaneous determination of caffeic acid, chlorogenic acid, and selected flavonoids further enables characteristic chromatographic profiles of *P. scutellarioides* extracts to be established and compared across experimental conditions [12,33,36,47,49,57,75,76].

In *P. scutellarioides*, HPLC-DAD/PDA has been used primarily for chromatographic profiling and analysis of phenolic constituents, whereas coupling liquid chromatography with MS or MS/MS provides greater confidence in metabolite annotation through molecular-mass and fragmentation information. This approach is particularly relevant to the chemically complex extracts of this species, in which compounds with similar chromatographic or spectrophotometric properties may coexist. LC-MS/MS has been applied to the characterization of anthocyanins, enabling cyanidin- and pelargonidin-derived glycosylated and acylated forms to be distinguished, and to the confirmation of rosmarinic acid through its characteristic fragmentation behavior. High-resolution MS and dereplication strategies further facilitate the recognition of previously reported metabolites and the prioritization of potentially novel constituents for subsequent structural investigation [33,37,39,42,45,48,49,61,75,79,80,81].

GC-MS studies of *P. scutellarioides* have revealed substantial variation in the volatile profile depending on plant material, cultivation conditions, and the procedure used to obtain the volatile fraction. Analysis of leaf essential oil obtained by microwave-assisted hydrodistillation identified 28 constituents, with spathulenol (24.57%), germacrene D (14.53%), bicyclogermacrene (11.24%), nonacosane (9.74%), and morillol (4.95%) as the predominant components [1]. More recently, the volatile profile of *C. scutellarioides* ‘Electric Lime’ was characterized using headspace solid-phase microextraction coupled with GC-MS (SPME-GC-MS) [34]. In the original study, “in vivo” material referred to plants grown under conventional cultivation conditions, whereas “in vitro” material referred to plantlets maintained under controlled tissue-culture conditions. Thirty volatile compounds were identified in conventionally cultivated plants, compared with 33 compounds in plantlets maintained under in vitro culture conditions. The total volatile-compound content increased from 2848.59 µg g^−1^ in conventionally cultivated material to 8191.47 µg g^−1^ in control in vitro cultures. Marked differences were also observed in the relative abundance of individual constituents: 1-octen-3-ol increased from 14.44% to 47.35% and 3-octanol from 4.79% to 15.19%, whereas copaene decreased from 18.44% to 5.36% and α-pinene from 15.63% to 6.87% [34]. Plant growth regulators further modified the volatile profile, with the highest total volatile-compound content reported for cultures supplemented with NAA (10,579.11 µg g^−1^) [34]. These findings demonstrate that cultivation conditions can markedly influence both the total abundance and relative composition of volatile metabolites in *C. scutellarioides*.

Structural studies of diterpenoids isolated specifically from *P. scutellarioides* have combined chromatographic isolation with MS and NMR spectroscopy to characterize several structurally distinct abietane derivatives. Three new abietane-type diterpenoids, spiroscutelones A–C, were isolated from the leaves of Indonesian *P. scutellarioides*, and their structures were established using spectroscopic analyses, including 1D and 2D NMR and high-resolution mass spectrometry, revealing an unusual structural architecture, particularly the cyclobutane-containing skeleton of spiroscutelone A [27]. A series of additional diterpenoids from the aerial parts of *P. scutellarioides*, including coleon O, coleon G, lanugone K, and fredericone derivatives, were characterized using chromatographic isolation followed by MS and NMR-based structural analysis [13]. In addition, a new abietane-type diterpenoid glucoside was isolated from the aerial parts of the species and structurally characterized using MS together with one- and two-dimensional NMR spectroscopy [15]. Collectively, these studies demonstrate that NMR and MS have been applied to *P. scutellarioides* primarily for the structural elucidation of isolated diterpenoids, enabling determination of their carbon skeletons, substitution patterns, glycosylation, and other structural features [13,15,27].

Beyond structural elucidation of isolated compounds, NMR-based metabolomic profiling has been applied to *P. scutellarioides* extracts to compare overall phytochemical patterns associated with cultivar, cultivation conditions, and extraction procedures. Combined with chemometric analysis, this approach enables differentiation of plant materials without requiring complete isolation of individual constituents and may therefore support comparative studies and quality control. FTIR provides complementary fingerprint information on major functional groups and can assist in rapid comparison of *P. scutellarioides* samples, although its limited molecular specificity prevents unequivocal identification of individual metabolites [4,10,37,82,83].

TLC and spectrophotometric assays have been used as complementary screening approaches in phytochemical studies of *P. scutellarioides* [4,32,39]. TLC provides rapid comparison of extract profiles and can support preliminary assessment of differences in phytochemical composition among plant materials and extracts [4,32,39]. Spectrophotometric assays have been applied to estimate aggregate parameters such as total phenolic and total flavonoid contents, providing useful comparative information on *P. scutellarioides* extracts obtained under different experimental conditions [4,35,44,73,83]. However, these approaches do not provide unequivocal identification or accurate quantification of individual metabolites; consequently, compound-specific characterization requires more selective chromatographic and spectrometric techniques [33,35,49].

Overall, comprehensive phytochemical characterization of *P. scutellarioides* requires the integration of complementary analytical techniques rather than reliance on a single platform. Preliminary screening and fingerprinting can guide subsequent targeted chromatographic analyses, while LC- and GC-based techniques should be selected according to metabolite polarity and volatility. For known metabolites, qualitative identification should be followed by quantitative determination using validated procedures and appropriate reference standards when available. In contrast, previously unknown metabolites require additional structural evidence, typically involving high-resolution mass spectrometry, compound isolation, and NMR spectroscopy. Thus, the analytical pathway should progress from preliminary profiling of crude extracts through chromatographic separation and tentative identification to quantitative determination or, when necessary, definitive structural elucidation [10,15,27,65,80].

## 5. Biological Activity

### 5.1. Antioxidant Activity

Antioxidant activity is among the most frequently investigated biological properties of *P. scutellarioides*, although the reported potency varies considerably depending on extract composition, solvent, cultivar, and assay conditions [10,19,67,72]. Quantitative DPPH analysis demonstrated an IC_50_ of 227.84 µg mL^−1^ for a 70% ethanolic leaf extract compared with 244.42 µg mL^−1^ for the corresponding aqueous extract [72]. Thus, the ethanolic extract exhibited moderately greater radical-scavenging activity, consistent with differences in the extraction of antioxidant constituents between the two solvent systems. Nevertheless, both extracts were substantially less active than the reference antioxidant vitamin C (IC_50_ = 7.27 µg mL^−1^) [72]. These quantitative findings indicate that the antioxidant potential of *P. scutellarioides* should not be inferred solely from the presence of phenolic compounds but should be evaluated in relation to extract composition, extraction conditions, and experimentally determined activity.

Quantitative comparison of antioxidant activity across studies remains challenging because different assays, extraction procedures, solvent systems, and reporting units have been employed [6,19,67,70,84]. Consequently, differences in reported antioxidant potency cannot be attributed exclusively to the phytochemical composition of *P. scutellarioides*, and direct numerical comparisons should be restricted to studies using comparable experimental conditions. The available evidence nevertheless indicates that extraction conditions and the resulting phenolic profile are major determinants of the measured antioxidant activity [19,67,70]. The type of solvent employed also has a significant influence [6,71]. Alcoholic extracts, particularly ethanolic and methanolic extracts, often exhibit high activity because of the efficient recovery of phenolic compounds [68,69,70]. Relationships between total phenolic or flavonoid content and antioxidant capacity have also been observed, although they are not always linear because of interactions among numerous metabolites [10,47,67,83,85,86,87,88].

Considerable differences may also occur among *P. scutellarioides cultivars* [10,19]. Intensely pigmented forms often contain higher concentrations of anthocyanins and other polyphenols, whereas green-leaved cultivars may exhibit different flavonoid profiles [10,19]. This variability indicates that genotype should be considered when comparing the antioxidant activity of individual extracts [10,63,67]. At the same time, the results of chemical assays such as DPPH, ABTS, and FRAP should not be directly equated with biological effects in living organisms [56,69]. Confirmation of actual cytoprotective properties requires further investigations using cellular, animal, and clinical models [4,38,56,86].

### 5.2. Antimicrobial Activity

Extracts and fractions of *P. scutellarioides* exhibit antimicrobial activity against Gram-positive and Gram-negative bacteria, as well as certain yeast-like fungi [10,11,34]. Experimental studies have evaluated their activity against, among others, *Staphylococcus aureus*, *Escherichia coli*, *Bacillus subtilis*, *Klebsiella pneumoniae*, and *Pseudomonas aeruginosa* [11,47]. In some cases, activity has also been observed against strains resistant to selected antibiotics, increasing interest in this species as a potential source of novel antimicrobial substances [52,56,69]. However, findings obtained in vitro require further confirmation before clinical applications can be considered [41,56].

Quantitative antimicrobial data further demonstrate substantial differences in susceptibility among individual microorganisms. For the ethanolic leaf extract, MIC values against the tested oral microorganisms ranged from 1.56 to 12.50 mg mL^−1^, whereas MBC values ranged from 3.13 to 200.00 mg mL^−1^. Among the Gram-positive species, *Streptococcus oralis* was the most susceptible, with an MIC of 1.56 mg mL^−1^ and an MBC of 3.13 mg mL^−1^, whereas *S. mitis* and *S. sanguinis* both showed MIC values of 6.25 mg mL^−1^. Considerably greater variability was observed in bactericidal activity against Gram-negative anaerobic species: *Aggregatibacter actinomycetemcomitans* showed an MIC/MBC of 3.12/6.25 mg mL^−1^, whereas *Treponema denticola* required 12.50 mg mL^−1^ for growth inhibition and 200.00 mg mL^−1^ for a bactericidal effect. These results demonstrate that describing the extract simply as “antimicrobial” obscures substantial organism-dependent differences in potency and further emphasizes the need to report quantitative endpoints rather than qualitative inhibition alone [11].

The mechanisms underlying antimicrobial activity are probably complex and depend on the predominant metabolites [1,47,48]. Terpenoids and volatile-fraction constituents may disrupt the integrity of microbial cell membranes, increase their permeability, and cause leakage of intracellular components [36,48,88]. Phenolic compounds may interact with proteins, enzymes, and cell-surface structures [48,52]. Abietane-type diterpenoids may also be particularly important because their reactive functional groups can facilitate interactions with cellular membranes and enzymes [36,69]. Flavonoids and synergistic interactions among individual extract constituents may additionally contribute to the overall antimicrobial effect [1,11,47].

The antimicrobial potential of *P. scutellarioides* has also been investigated in the context of dental applications [11,82,83,85,86,87,88,89,90,91,92,93,94]. Leaf extracts have exhibited activity against bacteria associated with oral and peri-implant diseases, including *Streptococcus mitis* and *Porphyromonas gingivalis* [11,89]. Antifungal activity has also been observed against *Candida* species, including *C. albicans* and *C. tropicalis*, potentially through disruption of cell membranes and enzyme function [10,11,34,95]. However, the potential use of such extracts requires evaluation of their safety, stability, and efficacy in more complex biological models [4,11,41,56].

Interpretation of antimicrobial findings also requires consideration of the methods employed [47,56,96]. Diffusion assays and MIC, MBC, and MFC values provide different types of information and should not be compared without accounting for the experimental conditions [11,36,56]. The lack of extract standardization also remains an important limitation, as preparations with substantially different chemical compositions may exhibit different activities even when tested using identical biological models [56,66,95].

### 5.3. Anti-Inflammatory and Anticancer Activities

The potential anti-inflammatory activity of *P. scutellarioides* has been associated with the presence of metabolites capable of modulating mediators of the inflammatory response [1,6,36]. Several extracts and isolated compounds have demonstrated the ability to reduce nitric oxide production and influence the activity of enzymes and signaling pathways involved in inflammation [4,38,86,96]. One of the proposed mechanisms involves modulation of cyclooxygenase (COX-1 and COX-2) activity, which participates in prostaglandin synthesis, as well as regulation of the NF-κB signaling pathway controlling the expression of numerous pro-inflammatory genes [6,52,97]. Suppression of NF-κB activation may consequently reduce the production of cytokines and other inflammatory mediators [95].

More direct quantitative evidence is available for diterpenoids isolated specifically from *P. scutellarioides*. Among the isolated compounds evaluated for inhibition of NF-κB activity, lanugone K showed the highest potency, with an IC_50_ of 4.5 µM, followed by 6-acetylfredericone B (IC_50_ = 9.7 µM), coleon G (IC_50_ = 11.0 µM), and coleon O (IC_50_ = 11.2 µM) [13]. These results provide compound-specific quantitative evidence supporting the anti-inflammatory potential of *P. scutellarioides* diterpenoids rather than inferring activity solely from the general phytochemical composition of the plant. Nevertheless, these findings originate from in vitro NF-κB inhibition assays and should not be interpreted as evidence of anti-inflammatory efficacy in vivo.

The anti-inflammatory effects may also be closely associated with antioxidant activity [4,10]. Oxidative stress and inflammation are interconnected processes, as excessive production of reactive oxygen species (ROS) can promote the activation of redox-sensitive signaling pathways, including NF-κB, thereby enhancing the expression of pro-inflammatory mediators [68,95,97]. Consequently, the antioxidant activity of phenolic compounds and flavonoids may contribute to attenuation of inflammatory signaling by limiting excessive ROS accumulation and subsequent NF-κB activation. Importantly, redox regulation may also provide a mechanistic link between the anti-inflammatory and anticancer effects reported for *Plectranthus*-derived metabolites. Whereas phenolic constituents may protect cells against excessive oxidative stress, selected diterpenoids can exert pro-oxidant effects in cancer cells, disturbing redox homeostasis and promoting mitochondrial dysfunction and apoptosis [53,60,97]. Thus, modulation of cellular redox balance may represent a common mechanistic element connecting the antioxidant, anti-inflammatory, and cytotoxic properties attributed to *P. scutellarioides* and related *Plectranthus* metabolites.

Quantitative cytotoxicity data obtained specifically for *P. scutellarioides* further strengthen the evidence for its antiproliferative potential. Among the abietane-type diterpenoids isolated from the leaves, spiroscutelone B showed the highest cytotoxic activity against the tested human cancer cell lines, with IC_50_ values ranging from 17.9 to 29.8 µM against MCF-7 breast, PSN-1 pancreatic, and HeLa cervical cancer cells, while exhibiting comparatively low cytotoxicity toward normal WI-38 lung fibroblasts [13]. In another study, coleon O isolated from *P. scutellarioides* showed IC_50_ values of 9.2 and 8.4 µM against human multiple myeloma cancer stem cells and RPMI 8226 cells, respectively [59]. These findings demonstrate that the antiproliferative potency differs substantially among individual diterpenoids and cellular models and indicate that biological activity should be evaluated at the compound level rather than attributed generally to the species or diterpenoid class. However, these IC_50_ values were obtained in in vitro cellular models and should be interpreted as measures of experimental cytotoxic potency rather than evidence of therapeutic efficacy in vivo.

However, the evidence should be interpreted according to its taxonomic origin. Direct quantitative data for *P. scutellarioides* remain considerably more limited than the broader evidence available for diterpenoids isolated from other *Plectranthus* species [13,53,55,59,60]. Therefore, mechanistic findings obtained for structurally related diterpenoids from other species can support biological plausibility but should not be considered direct evidence of anticancer activity of *P. scutellarioides*. This distinction is particularly important when discussing mitochondrial dysfunction, ROS generation, caspase activation, and apoptosis, for which much of the mechanistic evidence derives from compounds investigated across the genus rather than from standardized *P. scutellarioides* extracts [53,55,59,60].

## 6. Limitations of Current Research and Future Perspectives

The principal limitation of the current evidence on *P. scutellarioides* is the predominance of in vitro studies, including antioxidant, antimicrobial, enzymatic, and anticancer assays [4,36,62,86]. Animal investigations remain comparatively scarce, while clinical evidence for standardized extracts or isolated metabolites is very limited [10,62]. Consequently, the available data are insufficient to establish therapeutic efficacy, optimal dosage, long-term safety, or clinically relevant drug interactions [56,86].

Taxonomic identification remains an important limitation in studies of *P. scutellarioides*, particularly because the genera *Plectranthus* and *Coleus* have undergone substantial nomenclatural revisions and numerous ornamental cultivars differ in both morphology and chemical composition [34,52,56,86]. Misidentification may consequently lead to biological or phytochemical properties being attributed to the wrong taxon [10,56,67]. Future studies should therefore document the botanical identity and origin of plant material, deposit voucher specimens in recognized herbaria, and, where appropriate, incorporate molecular methods for taxonomic confirmation [10,47].

Another potentially important source of phytochemical variability is the timing of plant material collection. Although the available studies demonstrate that the accumulation of major metabolites in *P. scutellarioides* is influenced by genotype, cultivation conditions, light environment, and leaf temperature, directly comparable quantitative data concerning the effects of harvesting season, developmental stage, or plant age remain scarce [10,34]. Consequently, the current evidence does not allow a reliable assessment of how the qualitative and quantitative metabolite profile changes throughout plant development or across different collection periods. Future studies should therefore report key characteristics of the plant material, including the harvest season or collection period, developmental stage or plant age, and the specific plant part investigated, and should systematically quantify major phytochemical markers at different harvesting times using standardized analytical procedures. Such reporting would facilitate the identification of optimal harvesting conditions and improve the comparability and reproducibility of phytochemical studies.

The pharmacokinetics and bioavailability of *P. scutellarioides* metabolites remain insufficiently characterized [53,62,69]. High activity observed in vitro does not necessarily translate into therapeutically relevant exposure in vivo, particularly for poorly water-soluble diterpenoids [53,69]. Future studies should therefore determine the absorption, distribution, metabolism, and excretion of major bioactive constituents and establish whether biologically active concentrations can be achieved in target tissues [53,62].

Safety remains insufficiently characterized for *P. scutellarioides* preparations [48,97]. Preliminary reports of low toxicity for selected extracts do not establish long-term safety, and further studies should address acute and chronic toxicity, genotoxicity, mutagenicity, and potential organ-specific effects [56,62,66,97]. This issue is particularly important for diterpenoids and other metabolites exhibiting pronounced cytotoxic activity, for which pharmacological relevance depends not only on potency against cancer cells but also on selectivity over normal cells. Future cytotoxicity studies should therefore include appropriate non-cancerous cell models and report selectivity indices whenever possible [56,62].

The relationship between cultivation conditions and metabolite profiles also requires further investigation in *P. scutellarioides* [33,41,68]. Environmental factors, particularly light intensity, temperature, water availability, and mineral nutrition, may influence the accumulation of rosmarinic acid, anthocyanins, and other bioactive constituents [33,47]. Better control and reporting of these variables could improve both the reproducibility of phytochemical studies and the standardization of plant material intended for further biological or technological applications [33,47,78].

Future preclinical, clinical, and product-development studies should prioritize thoroughly characterized *P. scutellarioides* extracts or isolated metabolites with clearly defined phytochemical profiles. Standardization of extracts may be particularly important in translational studies requiring reproducible composition and dosing, whereas broader phytochemical investigations should focus on comprehensive chemical characterization rather than adherence to a single standardized extraction protocol. Particular attention should be directed toward establishing the pharmacokinetic, toxicological, and therapeutic relevance of the investigated preparations [4,33,48,66,86].

## 7. Discussion

Taken together, the available biological evidence suggests that the most informative interpretation of *P. scutellarioides* lies in linking specific phytochemical profiles with quantitative biological outcomes rather than considering broad activity categories in isolation. In particular, the pharmacological relevance of abietane-type diterpenoids should be evaluated in relation to both their cytotoxic potency and selectivity toward cancer versus non-cancerous cells, while the biological significance of phenolic-rich extracts should be interpreted in relation to their quantified phenolic composition. Such compound- and extract-level relationships are essential for distinguishing genuinely promising bioactive constituents from effects observed only in chemically undefined preparations. The current evidence therefore supports a transition from descriptive screening toward studies that directly integrate chemical composition, quantitative biological activity, and mechanistic validation. In this context, the principal value of future *P. scutellarioides* research will lie not in expanding the list of reported metabolites or biological activities, but in establishing reproducible quantitative relationships between defined plant material, extraction conditions, phytochemical composition, and experimentally validated biological effects.

## 8. Conclusions

The available evidence identifies *Plectranthus scutellarioides* as a chemically diverse species, with rosmarinic acid representing a major phytochemical marker and abietane-type diterpenoids showing particular pharmacological relevance. The strength of the available evidence, however, differs substantially among metabolite groups: rosmarinic acid is supported by quantitative extraction data allowing direct comparison of selected extraction conditions, whereas most diterpenoids and individual anthocyanins still lack cross-study comparable quantitative datasets. Extracts and isolated metabolites exhibit antioxidant, antimicrobial, anti-inflammatory, and cytotoxic activities; however, interpretation of these effects is limited by substantial variability in plant material, extraction procedures, phytochemical composition, and experimental models. Current evidence remains predominantly in vitro, while pharmacokinetic, safety, and clinical data are scarce. Future studies should therefore prioritize botanically and chemically characterized plant material, quantitative phytochemical profiling, comparable biological endpoints, and in vivo validation to establish reproducible relationships between composition and biological activity.

## Figures and Tables

**Table 1:** Influence of cultivar, geographical origin, and cultivation conditions on the phytochemical profile of *Plectranthus scutellarioides*.

Study	Plant Material/Cultivar	Origin/Cultivation Site	Factor Evaluated	Metabolites/Parameters	Main Findings
[6]	‘Color Blaze Dark Star’; ‘Trailing Queen’	Gowa/Sungguminasa and Makassar, South Sulawesi, Indonesia	Cultivar, geographical origin, extraction method	Alkaloids, flavonoids, tannins, phenols; extraction yield	Both cultivars contained the same major phytochemical classes, although extraction efficiency differed. The highest extraction yield was 35.8% for ‘Color Blaze Dark Star’ and 24.9% for ‘Trailing Queen’, indicating variability associated with plant material and extraction conditions.
[10]	‘Color Blaze Dark Star’; ‘Trailing Psycholeus’; ‘Trailing Queen’; ‘Beale Street’; ‘Trailing Rose’; ‘Flamingo’	Rantebelu Village, Luwu, South Sulawesi, Indonesia	Cultivar and extraction method	Total flavonoid content (TFC), TLC and GC-MS profiles	Clear cultivar-dependent differences were observed. TFC ranged from 1.667 to 4.305 mg RE g^−1^ DWE after maceration and 2.222–4.422 mg RE g^−1^ DWE after Soxhlet extraction. ‘Trailing Psycholeus’ showed the highest TFC after maceration, whereas ‘Flamingo’ showed the highest value after Soxhlet extraction.
[32]	Four accessions: coarse purple, fine purple, green and red leaves	Jumantono, Central Java, Indonesia	Accession and drought stress (watering every 1–4 days)	Total flavonoid content; TLC profile	Flavonoid accumulation depended on both accession and watering regime. Mean TFC values were 5.788, 7.813, 2.745 and 5.670 for coarse-purple, fine-purple, green and red accessions, respectively. The highest TFC (11.044) occurred in the fine-purple accession watered every 3 days.
[33]	‘Golden Dreams’	University Geisenheim, Germany	Light spectrum and leaf temperature (MPL, HPS, CDM and LED)	Rosmarinic acid (RA), total phenolics (TPC), total flavonoids (TFC), anthocyanins (TAC), luteolin and apigenin derivatives	Light conditions significantly affected metabolite accumulation. TPC was highest under CDM (66.8 ± 3.8 mg GAE g^−1^ DW) and LED (63.1 ± 5.2 mg GAE g^−1^ DW), and TFC was highest under CDM (16.2 ± 0.4 mg g^−1^ DW). RA and flavone glycosides accumulated preferentially under LED and CDM, which was associated with lower leaf temperature.

**Abbreviations**: CDM, ceramic discharge metal-halide; DW, dry weight; DWE, dry weight extract; GAE, gallic acid equivalents; HPS, high-pressure sodium; LED, light-emitting diode; MPL, microwave-powered plasma lamp; RA, rosmarinic acid; RE, rutin equivalents; TAC, total anthocyanin content; TFC, total flavonoid content; TLC, thin-layer chromatography; TPC, total phenolic content.

**Table 2:** Recommended analytical workflows for the qualitative, quantitative, and structural characterization of major metabolite groups in *Plectranthus scutellarioides*.

Target Metabolite Group	Initial Sample/Fraction	Primary Separation and Detection	Qualitative Identification	Quantitative Analysis	Structural Confirmation	Representative Applications
Phenolic acids and flavonoids	Polar/hydroalcoholic extract	HPLC-DAD/PDA	Retention time and UV-Vis spectra; LC-MS/MS provides precursor ions and diagnostic fragmentation patterns	HPLC-DAD/PDA or LC-MS/MS using authentic standards and calibration curves	LC-HRMS/MS for accurate mass and molecular formula; NMR when an unknown compound requires complete structural elucidation	Rosmarinic, caffeic and chlorogenic acids; flavonoids and their glycosides [12,33,42,45,47,75]
Anthocyanins	Acidified polar extract	HPLC-DAD coupled with LC-MS/MS	UV-Vis characteristics combined with accurate mass and MS/MS fragmentation enable differentiation of anthocyanidin aglycones and glycosylated/acylated derivatives	HPLC-DAD or LC-MS/MS using appropriate standards when available	HRMS/MS and NMR for previously uncharacterized derivatives	Cyanidin- and pelargonidin-derived anthocyanins [39]
Diterpenoids and other lipophilic terpenoids	Low- or medium-polarity fraction	LC/HPLC followed by MS or MS/MS	Molecular ions, accurate mass and fragmentation patterns permit dereplication and tentative assignment of known diterpenoids	HPLC/LC-MS when appropriate reference standards are available; otherwise quantitative comparison is limited	Isolation by preparative chromatography followed by 1D/2D NMR and MS/HRMS for definitive structure elucidation	Abietane-type diterpenoids and diterpenoid glucosides [13,15,27]
Volatile compounds/essential oils	Essential oil or volatile fraction obtained by distillation, extraction or headspace sampling	GC-MS	Retention behavior/retention indices and EI mass spectra compared with reference libraries and standards	GC-FID or GC-MS using calibration with appropriate standards	MS-supported identification; isolation and NMR only when unequivocal characterization of an unknown volatile is required	Mono- and sesquiterpenes and other volatile constituents [10,41,63,77,78]

## Data Availability

No new data were created or analyzed in this study. Data sharing is not applicable to this article.
